# Systematic review of sourcing and 3D printing: make-or-buy decisions in industrial buyer–supplier relationships

**DOI:** 10.1007/s11301-020-00198-2

**Published:** 2020-10-06

**Authors:** Matthias M. Meyer, Andreas H. Glas, Michael Eßig

**Affiliations:** Procurement Research Group, Bundeswehr University Munich, 85579 Neubiberg, Germany

**Keywords:** Procurement, Sourcing, Strategy, Additive manufacturing, 3D printing, Systematic literature review, M11, L24, L14

## Abstract

Additive manufacturing (AM) is regarded as a technology that has transformative and disruptive potential in nearly all industries. However, AM is not only about new production equipment and processes. Given the decreasing degree of vertical integration in many companies, suppliers add significant value to the finished product. AM might lead to the redesign of production networks, including a scenario in which the buyer uses AM to produce parts with data provided by suppliers. Overall, AM could have a major impact on the ways in which buyers and suppliers collaborate in the future. Nevertheless, research on AM in the field of industrial procurement remains scarce. This is surprising, given that AM is not only changing traditional procurement categories and creating new ones (comprising printers, powder raw materials, data and the associated engineering services) but AM’s widely discussed potential for decentralisation might also restructure the logistical aspects (transport, stocks) of supply chains. In addition, AM may resurrect the old procurement question of ‘make or buy’. Current research focuses on the logistical aspects of AM and concerns such issues of decentralisation (such as the diminishing need for transportation and the design of transport networks). In contrast, this research addresses the question of whether AM demands new answers to strategic sourcing questions. For this purpose, academic journal literature concerning procurement and AM search strings is reviewed. Selected articles are analysed using a fine-grained analytical framework of procurement strategies. The findings show that existing research lacks theoretical approaches and a systematic view of the topic. Specifically, the analysis reveals a number of distinct knowledge gaps, which present several potential directions for future research.

## Sourcing for additive manufacturing: setting the stage

**Fig. 1:**
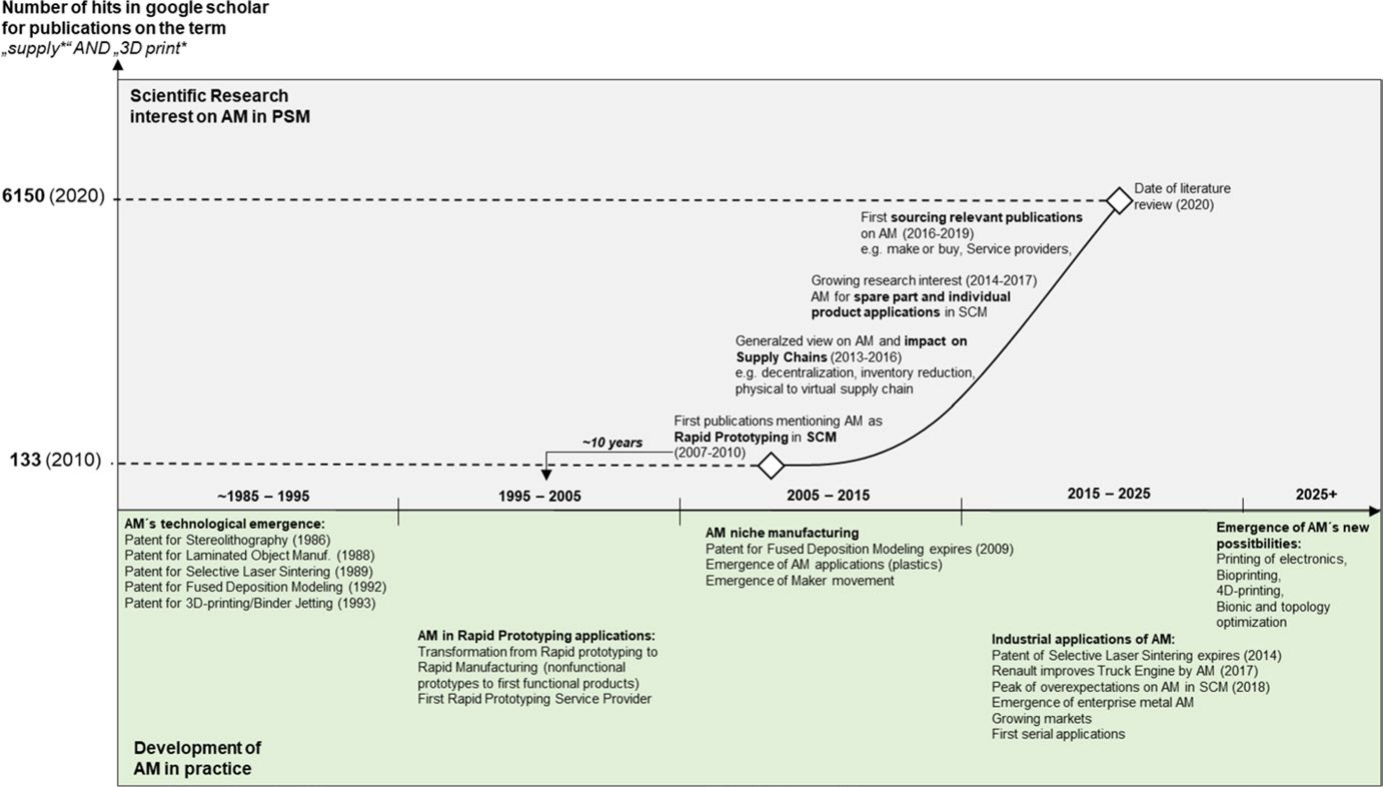
Emergence of AM in industrial practice compared to scientific research on PSM

In 1983, Chuck Hull developed stereolithography (SLA), the first additive manufacturing (AM) process. This is the starting point (see Fig. 1) of AM technology (Kietzmann et al. 2015). Within only 10 years, several more processes were invented and patented (Huang et al. 2013). AM has mostly been used for the production of nonfunctional prototypes, known as rapid prototyping (Ghadge et al. 2018). Due to the improved precision and quality of the produced parts (Khajavi et al. 2014) and the expiration of patents in 2009 and 2014 (Attaran 2017), 3D printers became more widely used and affordable (Kietzmann et al. 2015). Consumers increasingly adopted 3D printers for plastics, resulting in the maker movement, where consumers design and produce parts themselves (Waller and Fawcett 2014). Consumers therefore became potential competitors to established manufacturers (Nadkarni and Prügl 2020).

Furthermore, the increased production possibilities for plastics and the affordability of metal 3D printers led to the direct production of functional parts, known as rapid or direct manufacturing (Strong et al. 2018). Nowadays, AM is regarded as one of the main pacemaker technologies of the Fourth Industrial Revolution (Schweikl and Obermaier 2019).

Currently, in terms of Gartner’s hype cycle, AM in the field of supply chain management is classified as ‘minimally below the peak of inflated expectations’ (Gartner Inc. 2018). As a result, management consultancies predict that this technology will have a major impact on the market, such as annual growth rates of 35% (Roland Berger 2017) and a market value of US$250 billion by 2025 (McKinsey & Company 2017).

The ‘peak of inflated expectations’ warns that all forecast figures should be treated with caution. Nevertheless, numerous promising examples of industrial use already exist. For example, in 2017 Renault Trucks SAS introduced an improved truck engine, which had been developed by using the design and manufacturing benefits of AM. The number of parts was reduced by 25% (in total, 200 fewer parts were used), resulting in a weight reduction of 120 kg per engine and improving the transportation capacity for logistics providers (Renault Trucks SAS 2017).

Another example of AM’s industrial potential is the Ariane Project, a European joint venture to produce a commercially competitive carrier rocket. AM was used to optimise the injection head of the upper-stage engine, reducing the 248 parts of the injection head to a single part. This resulted in a 25% weight reduction and 50% cost reduction. In addition, the production time was decreased from three months to 65 h (EOS 2018).

These cases are an indication of AM’s relevance to manufacturing companies and industrial supply networks. AM’s importance will only increase as the technological possibilities increase, for example the 3D printing of electronics and biological structures. In fact, 4D printing, in which parts can change their properties, are becoming possible (Gartner Inc. 2018).

Research in procurement and supply management (PSM) addresses AM is at an early stage: The first scientific journal publication was in 2007 by Ruffo et al. This is despite the fact that new technologies are a main area of research in PSM (Hofmann et al. 2020). Nevertheless, early contributions, for example by Mohr and Khan (2015), assume that AM will impact on industrial companies and supply networks in several areas. These areas are improved product design and prototyping; improved production costs and flexibility (i.e., leading to mass individualisation); reduced production complexity; the possibility of production decentralisation; improved inventory and logistics; and altered legal and security-related aspects. The work of Mohr and Khan indicates that AM will change manufacturing processes from the early design stages to operations, including production, logistics and distribution.

The assumption can also be made that AM will affect PSM. First, manufacturing companies that use AM require new input categories, such as printers, raw materials in powder form, data, and services. Second, AM’s potential for decentralisation might raise the long-standing question of insourcing vs outsourcing (i.e., make or buy). Companies might therefore use AM as an enabler of new supply layouts. These new supply layouts could enable a smooth transition from the sourcing of goods to the sourcing of services. For example, buying the final AM output directly from the manufacturing service provider becomes an option (Rogers et al. 2016; Oettmeier and Hofmann 2016). This scenario demonstrates that AM can impact on sourcing decisions. Despite this, research in the field of PSM on the impact of AM is scarce and uncoordinated.

To address this shortcoming, this research study aims to generate a consolidated and comprehensive picture of the current state of scientific research in this field. The intention is to identify potential sourcing levers representing the input factor in order to develop a sourcing concept for AM. The following research question is therefore addressed: What is the status quo of scientific research in the field of ‘sourcing for AM’? To answer this question, we conducted a systematic multistage literature review (Easterby-Smith et al. 2015; Tranfield et al. 2003) with subsequent citation search (Wohlin 2014).

This paper is structured as follows: First, the basics of AM and sourcing are defined and merged into an analytical framework of sourcing for AM. Second, the methodology section presents the layout of the systematic review process, the filtering of results and a descriptive analysis. Third, the literature is analysed based on AM- and procurement-specific variables. Based on the results of the literature analysis, research gaps are identified and the potential effects of AM on sourcing are discussed. Our results provide answers to the questions posed by strategic sourcing levers, such as a shift to local and multiple sourcing. This sourcing levers represent the foundation for the formulation of a sourcing strategy for AM.

## Developing an analytical framework

### Additive manufacturing

**Fig. 2:**
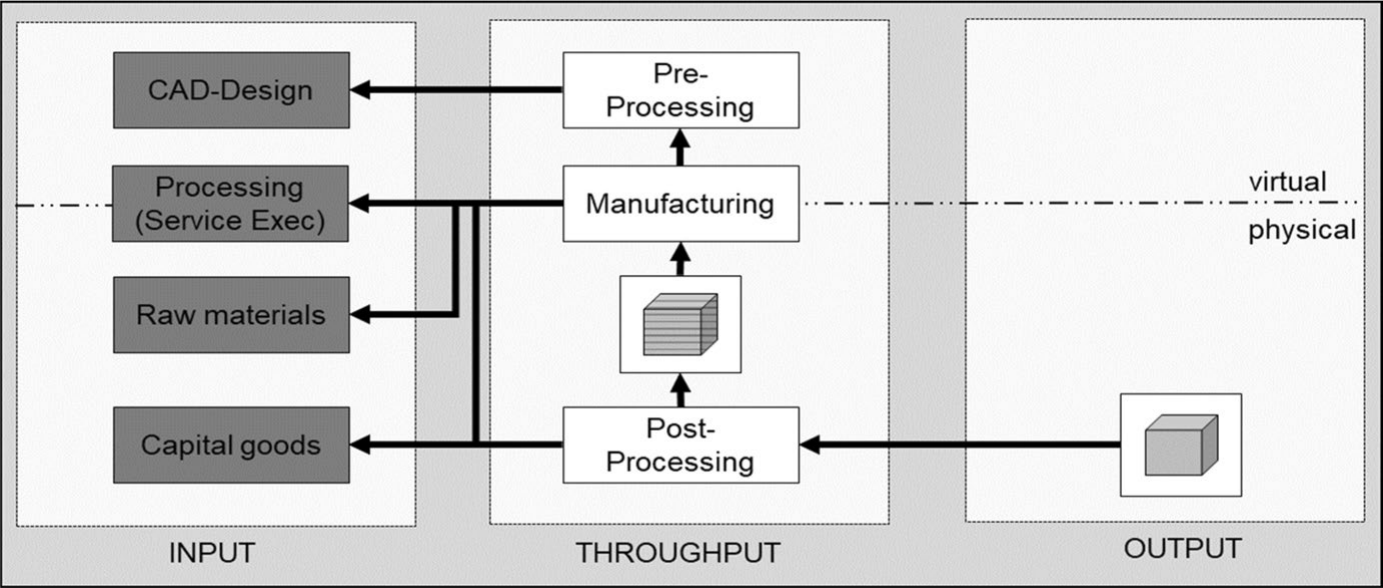
AM model

This section briefly introduces the technology of AM, its manufacturing process and specific characteristics. This description is then integrated into an AM model (Fig. 2).

Manufacturing can be classified into formative, subtractive and additive procedures (Gebhardt et al. 2016). AM stands for a technology consisting of not a single, but multiple distinctive manufacturing procedures (Durach et al. 2017). Layer manufacturing, generative manufacturing and 3D printing are synonyms for AM. 3D printing is the most commonly known, which originally only described the AM process of binder jetting. The distinctive AM procedure categories were normed by ISO/ASTM52900:2015, which is frequently cited by several publications (Durach et al. 2017; Khajavi et al. 2018a; Oettmeier and Hofmann 2016). These categories are binder jetting, directed energy deposition, material extrusion, material jetting, powder bed fusion, sheet lamination and vat polymerisation (ISO/ASTM 52900:2015 2015).

The definitions of AM given in the literature all refer to material that is positioned and joined layer by layer (Berman 2012; Beyer 2014) based on a digital representation of the object to be produced (Beyer 2014; Khajavi et al. 2014) until its physical reproduction is formed (Huang et al. 2013; Knofius et al. 2016).

A typical AM manufacturing process can be described as follows: A computer-aided design (CAD) file is created virtually. This file is converted into a sliced model that is readable by the 3D printer (Gebhardt et al. 2016; Gibson et al. 2015). Several pre-processing steps follow, such as adjusting the printer settings or preheating the build platform (Gibson et al. 2015). During the production process, layers of material are added iteratively until a physical part based on the virtual drawing is created (Gebhardt et al. 2016). When all the layers have been added, post-processing steps are done, such as the removal of support structures, heat treatment processes or surface finishing (Gibson et al. 2015).

All the above processes can be described in terms of the transformation process model, in which given input factors create a certain output (Slack et al. 2016). This perspective from the field of operations management helps to structure AM for a procurement perspective. All the processes require a certain set of input factors, which exist in either the virtual or the physical world. A virtual input factor for AM is the CAD model, which digitally describes the output to be manufactured (Rogers et al. 2016). Field service execution, or the personal execution of the process (Afshari et al. 2019), which includes software or manufacturing services, is relevant in both the virtual and the physical world (Rogers et al. 2016). Another input factor is the raw material (Khajavi et al. 2018b) for the printer, such as polymer or metal powders or filament. Capital goods involve not only the 3D printer (ISO/ASTM 52900:2015 2015) but also the machines required for post-processing activities (Strong et al. 2018). A basic AM framework is illustrated in Fig. 1, deviated by the process of Gebhardt et al. (2016). The figure shows the interplay of the virtual and physical world, which is one of the key characteristics of AM (Holmström and Partanen 2010; Huang et al. 2013). Figure 2 also references the generic transformation model, which is regularly used in operations management literature to describe processes (input–throughput–output).

### Sourcing in PSM literature

From 1985 until 2015, the vertical integration ratio for the share of supplier real net output in the automotive industry increased from 56 to 82% (Statista 2010). This trend of increased sourcing is accompanied by an increase in scientific research on the topic (Giunipero et al. 2019). Several publications (Carter and Narasimhan 1996; Roscoe et al. 2019) analysed and confirmed the influence of sourcing on the long-term performance of organisations.

**Fig. 3:**
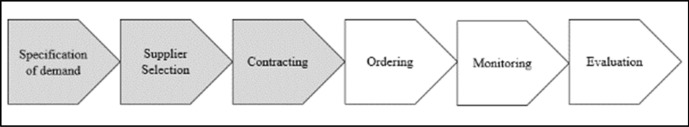
Sourcing within the procurement process based on van Weele and Eßig, 2017, p. 24

To understand the process of sourcing, the model that is used most often in procurement research (Bäckstrand et al. 2019) is that of Van Weele and Eßig (2017). This model describes sourcing as the creation of the best possible supplier strategy for a certain category of products. As a strategic part of the procurement process, sourcing involves demand specification (Anderson and Katz 1998), supplier selection and contracting (Van Weele and Eßig 2017, p. 22 ff), as illustrated in Fig. 3.

The decision to make or buy is the starting point and an important element in the sourcing process (Nikolarakos and Georgopoulos 2001; Tayles and Drury 2001; Vitasek 2016). When deciding whether a manufacturing process is to be executed in-house or performed by a supplier, the company’s strategy and the coordination of procurement with other functional strategies, such as logistics and production (Narasimhan and Carter 1998; Watts et al. 1995), should be considered. The ‘make or buy’ decision defines the number of value-adding activities that will be sourced from suppliers; in other words, it specifies the sourcing demand (Anderson and Katz 1998). On that basis, it is the task of procurement to satisfy the demand and to select and contract the right suppliers (Van Weele and Eßig 2017, p. 24) by matching these requirements to the intended supplier–buyer relationship (Hoque and Rana 2020).

Sourcing further requires the coordination of and an alignment between a firm’s strategy and functional strategies. Additionally, sourcing is characterised by the formulation and application of guiding governance rules in terms of the procurement strategy. Hesping and Schiele (2015) created a multilevel model to illustrate this scenario. The top level represents the firm’s strategy, from which functional strategies (level 2), category strategies (level 3) and sourcing levers (level 4) are derived. Sourcing levers (Hesping and Schiele 2015) or sourcing concepts (Arnold and Eßig 2000) are used to operationalise the category´s sourcing strategy and guide the efficient interaction with suppliers (Hesping and Schiele 2015). This includes strategic considerations, such as the geographic preference (local vs global), number of suppliers (single vs multiple sourcing), contract model and duration of the contract (Van Weele and Eßig 2017, p. 24).

However, sourcing tasks differ, and not every sourcing decision requires the same coordination effort. Choosing between straight rebuy, modified rebuy and new task buying is an important sourcing task. (Robinson et al. 1967). New task buying requires a great deal of information and coordination, whereas straight rebuy tasks require minimal information as the good has already been sourced. Modified rebuy tasks are between the two extremes (Robinson et al. 1967).

Overall, this research focuses on sourcing as a process that starts with the ‘make or buy’ decision, followed by demand specification, supplier selection and contracting. We acknowledge that sourcing requires strategic coordination on several levels and that there will be differentiation based on the nature of the buying task.

### Analytical framework: sourcing for AM

In this section, we derive the analytical framework for systematising the existing literature that deals with sourcing for AM (SfAM). The AM model (Sect. 2.1) is accordingly embedded into the sourcing model (Sect. 2.2).

First, we consider the AM model and descriptively analyse the throughput and output of AM. The AM procedure that is analysed in the literature is of interest, as is the purpose for which AM is used. AM output could be used for prototypes (Munguía et al. 2008; Pahwa et al. 2018; Ruffo et al. 2007), spare parts (Khajavi et al. 2018a; Li et al. 2017; Zhang et al. 2018), individual products (Chiu and Lin 2016; Christopher and Ryals 2014; Halassi et al. 2018), tools (Achillas et al. 2015; Attaran 2017; Huang et al. 2017) or serial parts (Minguella-Canela et al. 2017; Rylands et al. 2016).

First, we closely consider the different input categories and the ways in which these are analysed and treated in the literature.

Second, we look at the SfAM model. Here, the make-or-buy decision is the starting point and an integral element of the sourcing process (Vitasek 2016). That decision affects the other process steps, demand specification, supplier selection and contracting. Based on the make-or-buy decision, SfAM must consider the different categories of AM: CAD-design data, service processing, raw materials and capital goods.

Third, we regard SfAM as a strategic element. The SfAM model is therefore embedded in the model of coordination and strategic alignment across the strategic levels. Consequently, this review will analyze the literature based on the addressed levels of procurement strategy by Hesping and Schiele (2016).

**Fig. 4:**
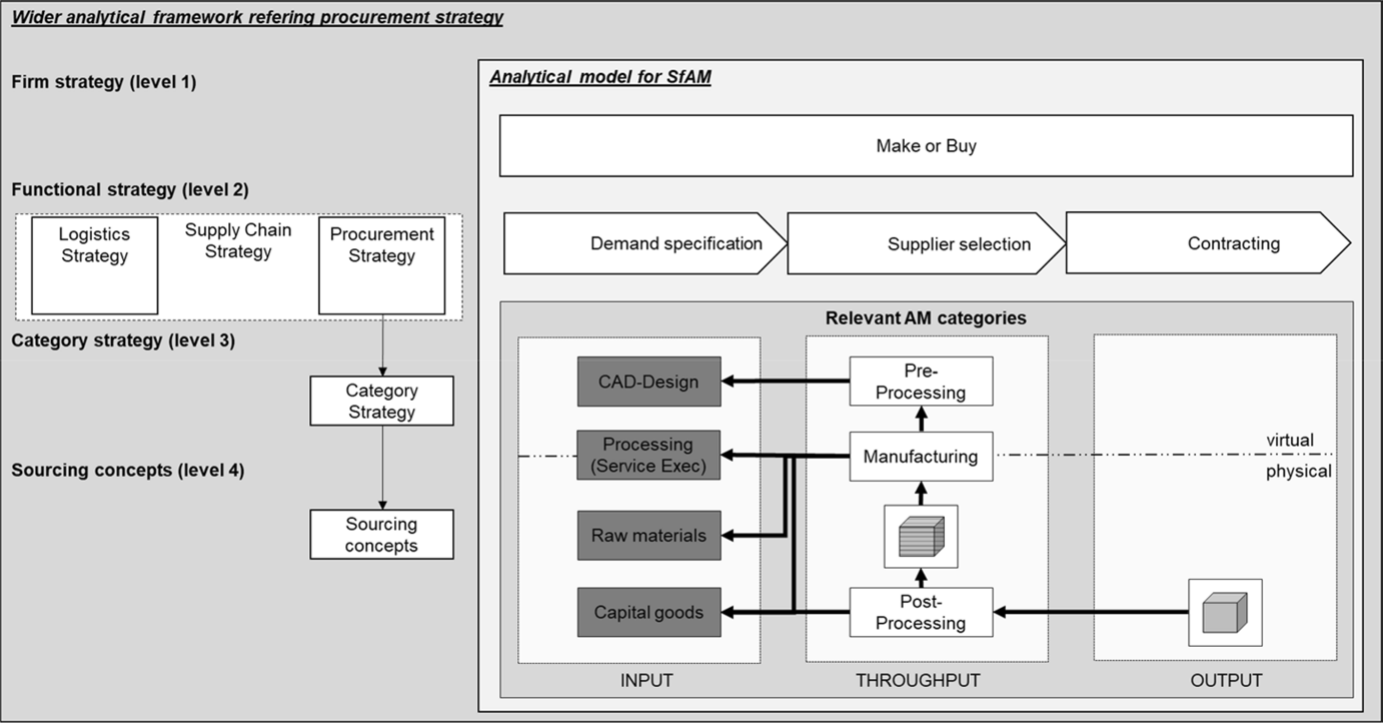
Analytical framework of the analysis

We also use other classification methods, such as the differentiation of sourcing tasks (Robinson et al. 1967) to explore SfAM in a more segmented way. Figure 4 illustrates the framework.

## Methodology

The following section deals with the procedure of the literature review, filtering and descriptive analysis of the found literature.

### Literature search

This research is based on a systematic multistage literature review to ensure completeness and reproducibility (Easterby-Smith et al. 2015; Tranfield et al. 2003). The goal is to identify papers at the point of intersection of AM and PSM, and relevant sourcing literature. Based on the findings of the literature research, a citation search (Wohlin 2014) was conducted to expand the pool of literature based on a preselected and included set of publications within the literature review. The inclusion criteria were peer-reviewed journal literature (or multiple citations within the identified set of literature) in English with a relevant contribution of AM in combination with PSM.

The search string therefore consisted of the term ‘sourcing’, its synonyms or related terms, connected via a Boolean OR function, plus AM and its synonyms. Both terms addressing PSM and AM were connected via a Boolean AND function, as indicated here: (“Sourc*” OR “Procure*” OR “Purchas*” OR “Supply*” OR “Acquisition”) AND (“Additive Manufacturing” OR “Rapid Prototyping” OR “3d print*” OR “Direct Digital Manufacturing”). The search was conducted within the databases of *EBSCOHost*, *EmeraldInsights*, *IEEE xplore*, *JSTOR ScienceDirect*, *Sage*, *Scopus, Taylor and Francis* and *Wiley InterScience*.

### Filtering and descriptive statistics

**Fig. 5:**
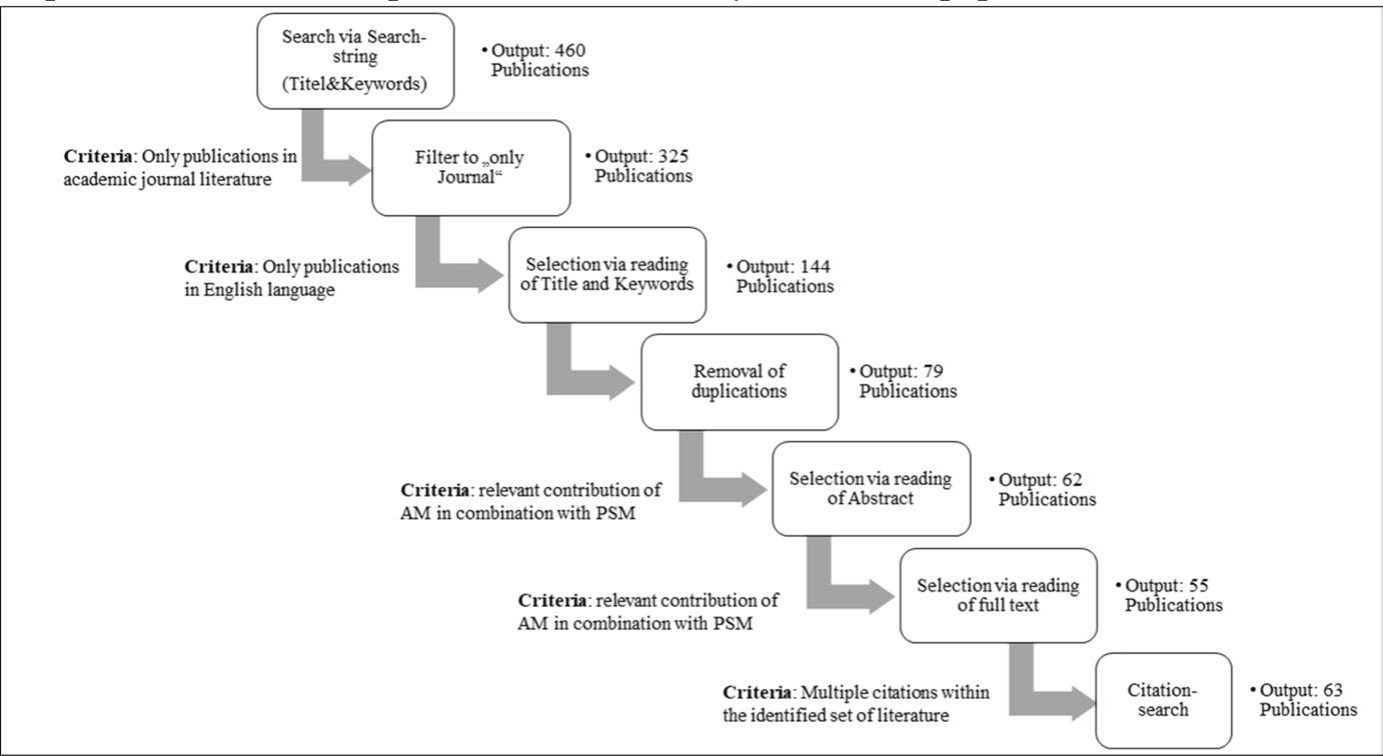
Procedure of the systematic literature review

The initial database search, which considered the titles and keywords of the research articles included within the search string, resulted in an output of 460 publications, as seen in Fig. 5. This was filtered by including only peer-reviewed journal literature in English, reducing the output to 325 articles. Filtering by screening the article’s title and keywords reduced the number to 144. When duplications were removed, 79 publications remained. Further selection was done by reading the abstract (output 62) and full text (output 55). By executing backwards snowballing, the number increased to a final output of 63. This set of publications was analysed for this paper.

The distribution of publications by journal shows a diversified picture, as seen in Table 1. The methodology used was mostly conceptual (38.1%), followed by case study research (31.7%) and modelling (23.8%). This confirms the need for empirical research.

**Table 1:** Distribution by journal ranking, research methodology and industry sector

Journal	No.	%
Journal of Manufacturing Technology Management	6	9.5%
Int. Journal of Physical Distribution & Logistics Management	6	9.5%
Rapid Prototyping Journal	5	7.9%
Journal of Operations Management	4	6.3%
Additive Manufacturing	3	4.8%
International Journal of Production Research	3	4.8%
Computers in Industry	3	4.8%
Business Horizons	2	3.2%
Computers and Industrial Engineering	2	3.2%
International Journal of Production Economics	2	3.2%
Journal of Business Logistics	2	3.2%
Journal of Industrial Ecology	2	3.2%
Journal of Manufacturing Systems	2	3.2%
Production Planning & Control	2	3.2%
Sum of single articles in a journal	19	30.4%
*Research methodology*		
Conceptual	24	38.1%
Literature Analysis	6	9.5%
*Qualitative methods*		
Interview	5	7.9%
Case study	20	31.7%
*Quantitative methods*		
Modelling	15	23.8%
Survey	8	12.7%
Experiment	1	1.6%
Simulation	2	3.2%
*Branch focus*		
No specific industry focus	43	68.3%
Aerospace	11	17.5%
Automotive industry	6	9.5%
Medical	6	9.5%
Consumer goods industry	4	6.3%
Engineering industry	4	6.3%
Food industry	1	1.6%

Furthermore, most of the articles did not have an explicit industry focus (68.3%), while some articles had a distinct focus on aerospace, automotive and medical applications. The consumer goods, engineering and food industries were also mentioned.

Articles that cover relevant aspects of PSM in the context of AM can be found in journals that specialise in SCM-related topics, such as the *International Journal of Physical Distribution & Logistics Management*. However, such articles also appear in journals that cover general business-related topics, such as *Business Horizons*, and manufacturing-oriented journals, such as the *Journal of Manufacturing Technology Management*. This spread of journals suggests that literature on PSM in the context of AM has not yet been consolidated and is strongly driven by manufacturing and logistics related journals. The main journals covering PSM-specific topics, such as the *Journal of Purchasing and Supply Management* or the *Journal of Supply Chain Management*, contain not a single publication on this technology.

**Fig. 6:**
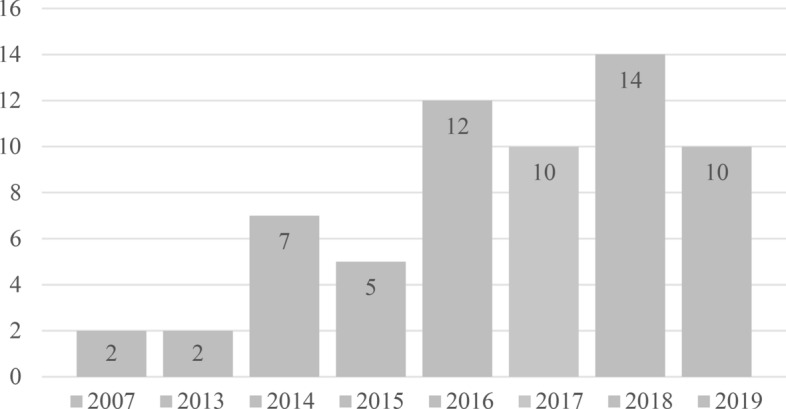
Distribution of journal papers by year of publication

All publications analysed were published between 2007 and 2019. Most were published from 2016 to 2019, as seen in Fig. 6. A possible reason for this increase in publications is that the patent for selective laser sintering (Deckard 1994) expired and the price of industrial 3D printers came down, raising industrial and research interest in the topic.

The geographical distribution by research location of the author shows research hubs on SfAM in the United Kingdom (19 publications), followed by the United States (14), Finland (10) and Germany (nine). The remaining research locations are positioned in Europe, Asia and Australia. No research on the topic was found on the continents of South America and Africa.

## Results of the literature review

### AM focus—materials and procedures

The technology ‘AM’ describes not one general but a set of multiple manufacturing processes that coexist (Durach et al. 2017). As stated, ISO/ASTM52900 (2015) classifies AM into seven process categories. Each process has its advantages, weaknesses (Huang et al. 2013) and fields of application (Holmström et al. 2016). It is surprising, therefore, that most of the literature on sourcing in the context of AM generalises about AM and does not mention specific AM processes, as seen in Table 2.

**Table 2:** Distribution of publications based on addressed AM-processes and materials

AM-processes addressed	
Unspecific	79.7%
Powder bed fusion	25.4%
Material extrusion	8.5%
Material jetting	8.5%
Vat polymerisation	8.5%
Binder jetting	6.9%
Direct energy deposition	1.7%
Sheet lamination	1.7%
*Materials addressed*	
Unspecific	79.7%
Metal	18.6%
Polymer	16.9%
Photopolymer	3.4%

This shows that current research on SfAM lacks specialisation. The process that gets the most explicit attention is power bed fusion (PBF), which can be used to manufacture plastics, metals, and ceramics (Holmström et al. 2016). This is followed by material jetting (plastics and metal), binder jetting (metals, ceramics, glass) and material extrusion (plastics), vat polymerisation (photopolymers), sheet lamination (metals, paper) and direct energy deposition (metals). However, the discussion takes place in only a few research studies. Most paper papers do not explicitly mention a specific AM procedure.

In addition, articles do not explicitly mention a specific material, and the majority fail to even mention the material (79.7%) (see Table 2). There is an equal distribution of papers that address metals and polymers, even though manufacturing with polymers has reached a higher level of technological maturity than metals (Mellor et al. 2014). The reason could be that there are more industrial applications for 3D printing metals than plastics. Photopolymers are addressed in only 3.4% of the publications and seem to be less important in the existing areas of application.

As the previous two sections of this study have demonstrated, current research addresses AM in generalised terms. Specific information on AM processes, their corresponding materials and fields of application is not provided or considered.

### How does AM affect PSM?

**Fig. 7:**
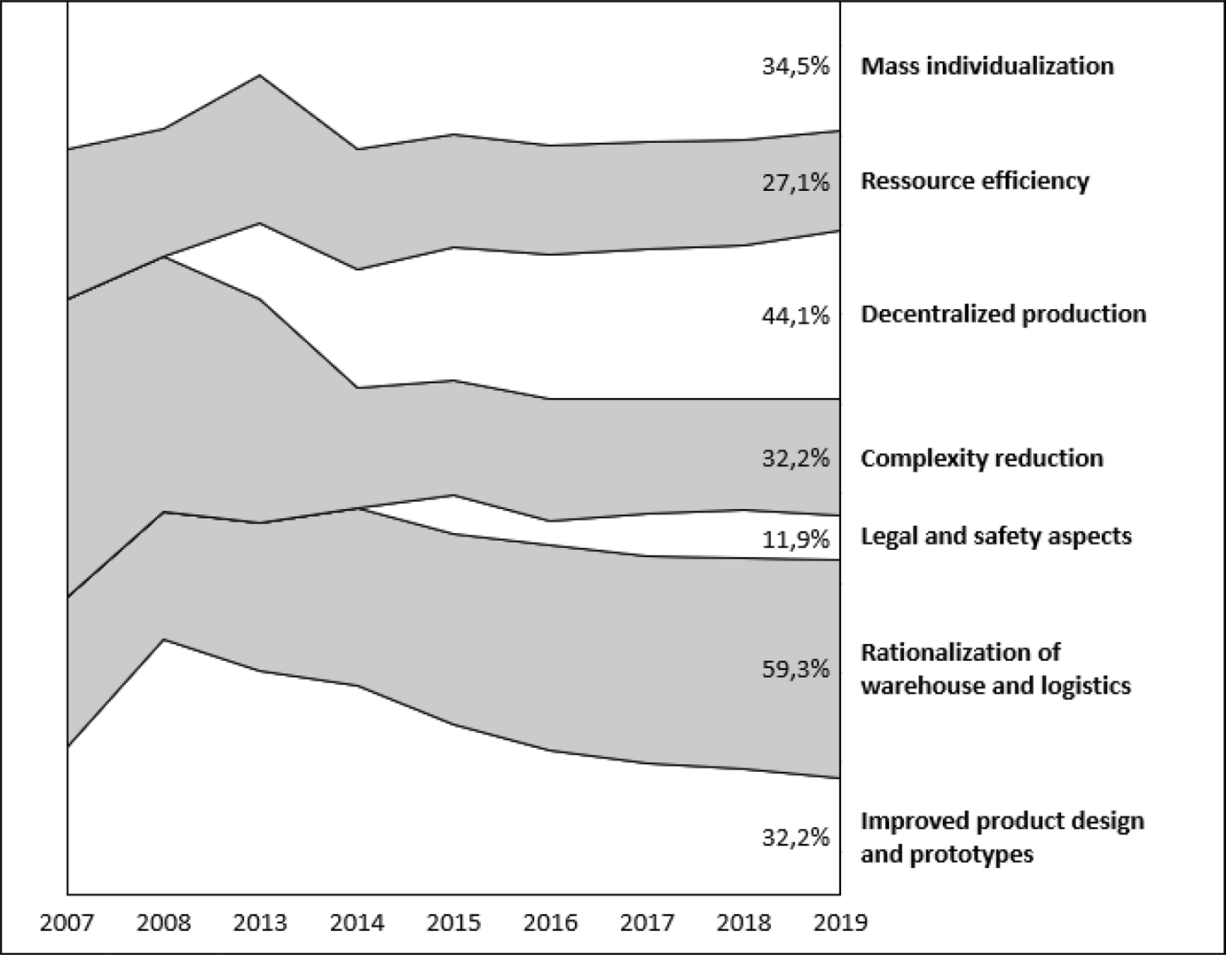
Percentage of publications addressing effects of AM on SCM over time

In this section, we classify the literature according to AM’s effects on PSM. The starting point is the work of Mohr and Khan (2015), which classifies these effects into seven categories. These are the rationalisation of warehousing and logistics, increased decentralised production, mass customisation of products, reduction of complexity, improvements in product design and rapid prototyping, a more efficient use of resources, and consequences for legal and safety aspects. This categorisation was used to classify journal literature based on the research focus, as seen in Fig. 7. The topic that received the most attention (35 articles) is AM’s effect on warehousing and logistics. Research on this topic includes work on decreasing inventory levels (Attaran 2017; Cohen 2014; Ghadge et al. 2018; Knofius et al. 2016; Muir and Haddud 2017; Pour et al. 2017), the possibility of ordering smaller batch sizes (Attaran 2017; Pour et al. 2017) and the reduction of transportation in the supply chain (Attaran 2017; Barz et al. 2016). Other research papers address the implications for logistics service providers (Öberg 2019) as supply chains become increasingly dematerialised (Chekurov et al. 2018; Tuck et al. 2007).

The topic that received the second most attention (26 publications) is the shift to decentralised production. This refers to manufacturing being located closer to the origin of demand, for example through AM production hubs (Minguella-Canela et al. 2017; Öberg 2019; Ryan et al. 2017; Strong et al. 2018, 2019), mobile AM manufacturing (Ryan et al. 2017), or directly at the customer’s location (Attaran 2017; Braziotis et al. 2019; Khajavi et al. 2014; Li et al. 2017; Minguella-Canela et al. 2017; Ryan et al. 2017) with the possibility of the end customer becoming the producer (Halassi et al. 2018; Kietzmann et al. 2015; Minguella-Canela et al. 2017; Öberg 2019; Ryan et al. 2017). These possibilities bring remote areas, such as humanitarian aid projects or war scenarios, into the picture (de La Torre et al. 2016).

Twenty publications addressed the increased individualisation of products, which has the effect of increasing a customer’s involvement in the design process. Customers either design the product themselves (Chiu and Lin 2016; Waller and Fawcett 2014) or they do so in combination with the manufacturing firm (Oettmeier and Hofmann 2017; Shukla et al. 2018). Such products could find wide-ranging application in the medical industry, where a high degree of customer adjustment is required, for example prostheses (Emelogu et al. 2016) and hearing aids (Oettmeier and Hofmann 2016) In the case of individualized products, the customer order decoupling point is expected to move closer to the end customer (Minguella-Canela et al. 2017; Ryan et al. 2017) in the supply chain.

Nineteen publications addressed the effect of complexity reduction. This means that in single products the amount of parts is reduced (Attaran 2017; Togwe et al. 2018; Tuck et al. 2007) Parts consisting of multiple functionalities can get consolidated into a single part, without the need for further assembly (Barz et al. 2016; Cohen 2014). Complexity reduction also means that certain steps within the supply chain become redundant or they are simplified (Attaran 2017; Ivanov et al. 2019; Khajavi et al. 2018b; Sasson and Johnson 2016), for example the production of tooling (Cohen 2014; Romero-Torres and Vieira 2016).

The same number of publications dealt with improvements in product design and rapid prototyping. This is the area in which AM was first applied, but it declined over the years as new fields of application opened up. AM enables the creation and manufacturing of complex designs (Cohen 2014), such as cavities or lattice structures (Beyer 2014), or software-based improvement of products (Holmström and Gutowski 2017), such as topology or bionic optimisation (Beyer 2014). These capabilities result in designs and products that are lightweight (Beyer 2014) or that cannot be produced by traditional manufacturing processes. This leads to new approaches, such as ‘design for AM’ (Chiu and Lin 2016). These developments makes it easy to make and test prototypes (Munguía et al. 2008), shortening the time to market and lowering product development costs (Attaran 2017).

Sixteen publications address the topic of increased resource efficiency due to AM. Waste during manufacturing is reduced because material is only applied where required, and products are only produced when required (Holmström and Gutowski 2017). This effect is linked to improved lightweight design (Barz et al. 2016). Furthermore, decentralised production and the associated elimination of transport reduces carbon dioxide emissions (Holmström and Gutowski 2017; Huang et al. 2017), which could be used for concepts of a more sustainable sourcing. Spare parts can easily be produced in small quantities, which reduces obsolescence and increases the lifespan and usage of a product (Chekurov et al. 2018; Holmström and Gutowski 2017).

The impact of AM on legal and safety aspects in the supply chain is addressed by 11.9% of the publications, making it the area least discussed. Topics raised are intellectual property issues (Dwivedi et al. 2017; Kietzmann et al. 2015; Shukla et al. 2018), the need for AM quality control due to sample size one (Kietzmann et al. 2015; Togwe et al. 2018) and the threat of open-source designs, for example the possibility of manufacturing weapons (Attaran 2017).

The effects discussed above refer to changes in the interaction with suppliers, which demand new sourcing concepts. The legal implications will also require new contrac-tual models. However, research on this topic is still scarce.

### A theoretical perspective of AM in PSM

This section reviews literature based on the theoretical approach. Our investigation of journal literature revealed that more than 90% of publications (see Table 3) did not adopt a theoretical approach. This is surprising, given that SfAM is a relatively recent phenomenon and that conceptual/theoretical work lays the foundation for empirical work. Non-theoretical research work dominated, and most papers employed an empirical approach.

**Table 3:** Distribution of publications by theoretic approach

Publications without theoretic approach	n						%				
	54						91.5%				
Publications using a theoretic approach	9						15.3%				
	Theories used in the publications										
Publication using a theoretic approach: (sorted by year of publication)	Transaction cost theory	Resource-based view	Knowledge-based theory	Contingency theory	Resource dependency theory	Agency theory	Institutional theory	Network theory	Dynamic capabilities	Unified theory of accept. and use of techn.	Systems theory
Ruffo et al. (2007)	x	x	x		x						
Waller and Fawcett (2014)	x	x		x	x	x	x	x			
Thomas (2016)		x							x		
Muir and Haddud (2017)									x		x
Ghadge et al. (2018)				x							x
Halassi et al. (2018)										x	
Öberg (2019)					x						
Hedenstierna et al. (2019)	x										
Roscoe et al. (2019)			x								

The remaining 10% of publications reviewed one or multiple theories. The first theoretical contribution was made by Ruffo et al. (2007). They mainly used the knowledge dependency theory and the resource dependency theory for the make-or-buy decision in AM, which causes either capacity or knowledge constraints. In addition, transaction cost theory and the resource-based view were referred to but not elaborated on.

In an editorial for the *Journal of Business Logistics*, Waller and Fawcett (2014) appealed for more theory-driven research of AM in SCM and proposed several theories as a possible foundation. The theories include transaction cost economics, a resource-based view, contingency theory, resource dependence theory, agency theory, institutional theory and socio-technical theory. Subsequently, seven more theoretical contributions were published between 2014 and 2020, mostly employing the resource-based view, resource dependency theory and transaction cost theory.

Apart from the external grand theories (EGTs) of PSM (Spina et al. 2016), more unusual theories were applied, such as the unified theory of acceptance and systems theory. Although many publications dealt with the effects of AM on supply chains, network theory was chosen by only one researcher. Overall, this lack of theoretic approaches reveals a major research gap and suggests a direction for future research.

Classifying the research according to theoretic approach shows that most publications considered the effects of AM from either the end-user or the buyer’s perspective (51%). This includes publications on manufacturing firms implementing AM. The network view (41% of publications), which considers influences on the whole supply chain, is the second most represented point of view. The perspective of the seller (10%) or a dyadic approach (9%) is rarely used.

### Content analysis according to buying task and product type

**Fig. 8:**
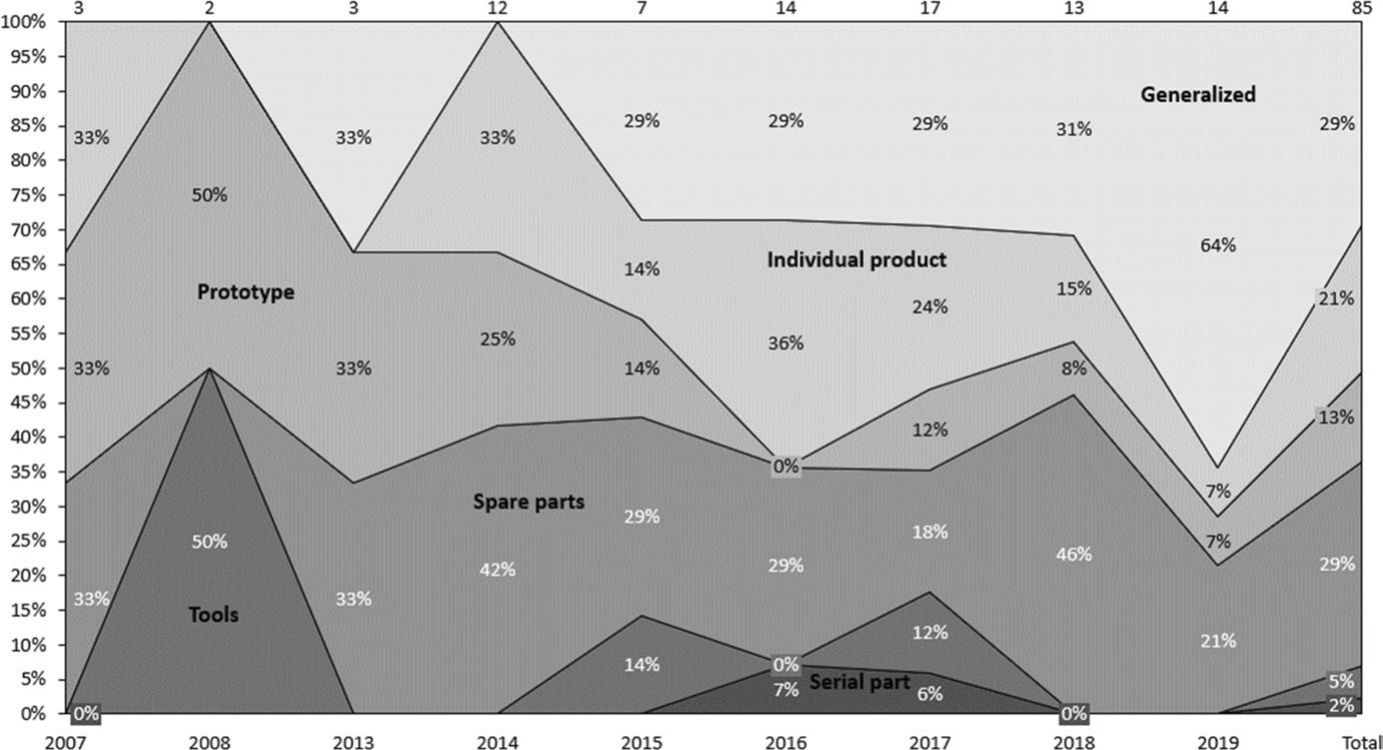
Classification of AM product type

In this section, publications are classified according to the product type to be manufactured by AM and demanded by the internal customer within the buying organization. As illustrated in Fig. 8, 29% of publications did not address a specific AM output category. Another 29% dealt with the production of spare parts (rapid sparing), or individual products (21%), which implies that researchers see the greatest potential for these product types being manufactured by AM. The production of AM to produce prototypes (rapid prototyping) is addressed in 13% of publications but diminished over the years. The production of tools (rapid tooling) was addressed in 5% of publications, peaking in 2008. Thereafter, it was rarely addressed. The fewest publications (2%) dealt with AM for serial products.

Analysing the literature according to the buying task indicates that mostly straight rebuys, such as the buying of spare parts, or modified rebuys, such as individual products, are addressed. Hereby the make-or-buy question has not to be asked again. New task buying, where the fundamental make-or-buy question is asked, is seldom discussed. Additionally, AM serial parts are never the focus of research focus, perhaps because of AM’s current technological benefit, which is to make products in small numbers (Oettmeier and Hofmann 2016).

### Content analysis: AM in the wider analytical framework of procurement strategy

**Fig. 9:**
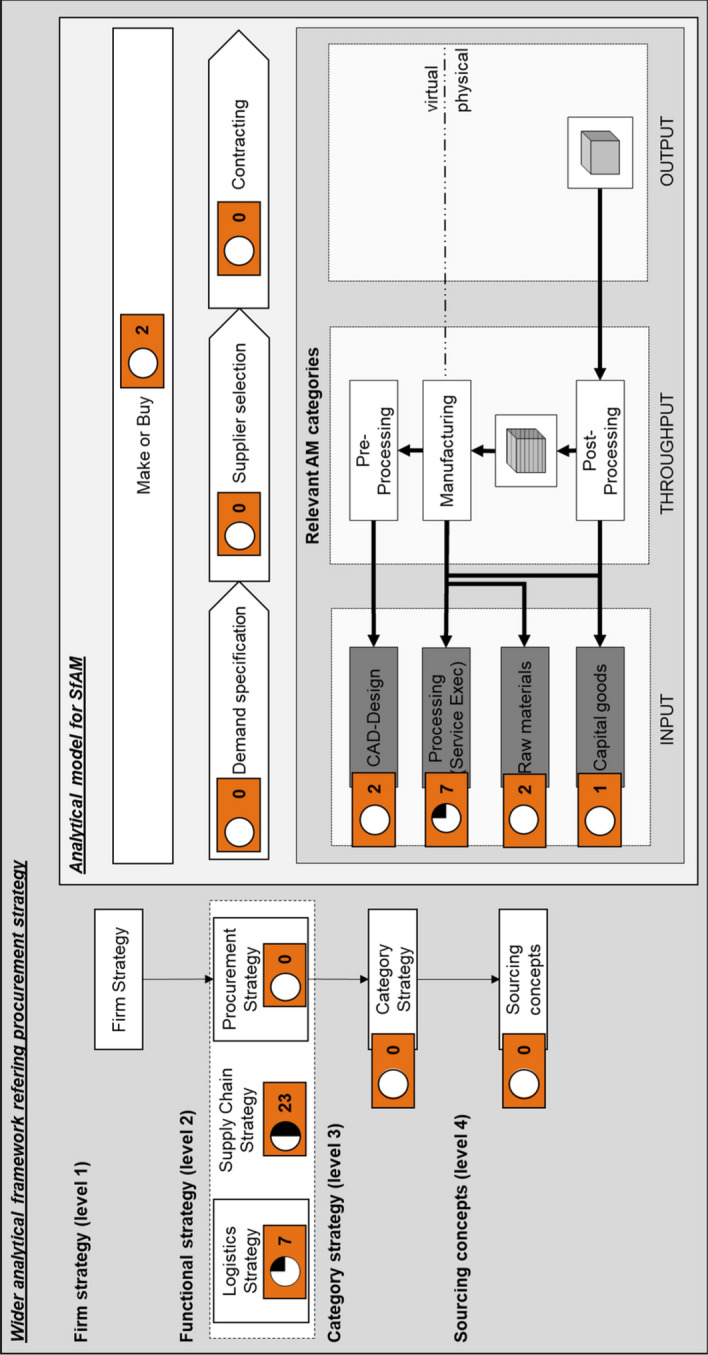
Publications classified in the analytical framework of SfAM

The functional strategy level is analysed first. Figure 9 shows the results within the analytical framework, indicating the current status of discussion in journal literature. The figure in the orange box indicates the number of publications that explicitly address the associated topic, whereas the Harvey ball represents the status in current research. Based on this, research gaps and areas for further research will be identified in the following section.

Twenty-three publications explicitly covered an AM-related supply chain strategy, which is located above the sourcing strategy on the functional strategy level. Tuck et al. (2007) stated that AM produces benefits for both supply chain configurations – lean and agile (Fisher 1997). In lean supply chains, AM should be used to reduce waste, costs and time (Romero-Torres and Vieira 2016; Tuck et al. 2007). In agile supply chains, flexibility (Oettmeier and Hofmann 2016; Tuck et al. 2007) and fast reconfigurability are the focus (Tuck et al. 2007).

Another supply chain strategy that is discussed is whether to implement AM in a centralised or decentralised configuration (Bogers et al. 2016; Braziotis et al. 2019; Emelogu et al. 2019; Khajavi et al. 2014, 2018a; Li et al. 2017; Liu et al. 2014; Minguella-Canela et al. 2017) or even in a hub configuration (Khajavi et al. 2018a; Ryan et al. 2017). The benefits of a centralised configuration, on the one hand, is the increased use of the printers (Braziotis et al. 2019). In addition, personnel and machinery costs will be lower, but transportation costs will increase (Braziotis et al. 2019; Khajavi et al. 2014, 2018a). Greater controllability of quality is also a benefit (Braziotis et al. 2019). On the other hand, the decentralised approach has a short response time, high costs for machinery and personnel, but low transportation costs (Braziotis et al. 2019; Khajavi et al. 2014, 2018a). If the average demand is too low (Liu et al. 2014), capacity utilisation will drop (Braziotis et al. 2019). A hub configuration would only apply for printers that are not capital intensive and have a high production rate (Khajavi et al. 2018a). Clearly, capacity utilisation of printers in response to demand strongly influences the manufacturing location and supply chain configuration (Emelogu et al. 2016; Khajavi et al. 2018a).

Another consideration is whether to implement AM with a standalone configuration or to combine it with traditional manufacturing processes (Braziotis et al. 2019; Chiu and Lin 2016; Rylands et al. 2016; Strong et al. 2018, 2019). Researchers (Emelogu et al. 2016, 2019; Strong et al. 2018, 2019) have used a mathematical model to identify the best location for AM hubs. Christopher and Ryals (2014) predicted that the supply chain will become more customer driven and evolve to become a demand chain, where AM gives more power to the consumer. This notion corresponds with the findings of Oettmeier and Hofmann (2017), who showed that the adoption of AM is strongly driven by the demand side of the supply chain.

Minguella-Canela et al. (2017) stated that the correct use of AM depends on the degree of individualisation and whether the demand for an output is stable or unitary. This classification provides first indications for procurement by which constellations e.g. buying of additional capacity from suppliers for the absorption of unitary demands as well as the plannability of raw material usage. AM should be used to source seasonal products with a low lifespan, whereas standard product products with a unitary demand with long lead times should be sourced by traditional manufacturing. For more customised products, AM should be used for postponement of production in supply chains (Minguella-Canela et al. 2017; Zhang et al. 2018), such as manufacturing individual products for unitary demands or providing optional individual components for a standard system by stable demands (Minguella-Canela et al. 2017).

Oettmeier and Hofmann (2016) suggested that AM should first be implemented for low-volume individual products, because the high unit costs will make it economically feasible. Öberg (2019) clustered the participants and their individual interests together in an AM supply chain. Chekurov et al. (2018) and de La Torre et al. (2016) introduce an implementation plan for the manufacturing of spare parts. Even if products manufactured by AM cost more than products made by traditional processes, costs can be compensated by reduced transportation and inventory (Chekurov et al. 2018). This is the area in which AM’s potential for producing spare parts in humanitarian aid missions comes to the fore, because the physical supply of spare parts from outside is hardly possible in these situations (de La Torre et al. 2016).

Seven publications suggest an AM logistics strategy located on a functional strategic level, which can serve as a reference point for procurement due to the strong interconnection of logistics and procurement on a functional level. Ghadge et al. (2018) modelled an aircraft supply chain, demonstrating that AM increases the service level within logistics processes of organisations by downsizing inventory (Liu et al. 2014) without including a cost calculation. The modelled AM scenario was not as vulnerable to demand fluctuations as the scenario using traditional manufacturing methods.

The model by Zhang et al. (2018) showed that an AM warehousing strategy still lacked cost competitiveness when compared to traditional methods. Heinen and Hoberg (2019) analysed the existing inventory of a manufacturing company to determine which parts could profitably be produced by AM. The result showed that around 8% of the total inventory could be switched to AM on-demand production (Heinen and Hoberg 2019). De La Torre et al. (2016) suggests that a logistics strategy for humanitarian aid missions should build a redundancy of AM and traditional suppliers as not all demands can be manufactured solely using AM.

For the level of an AM category not a publication considering strategy topics was found.

Similarly, sourcing levers and sourcing concepts did not feature as research topics. Rogers et al. (2016) addressed the neglect of these topics, suggesting the following as future research questions: ‘What type of sourcing (single vs multiple; local vs global) should be adopted?’ ‘What type of relationship is most appropriate for the various 3D printing services?’

### Content analysis: an analytical model for sourcing for AM

The make-or-buy decision is the starting point and an integral element of sourcing. As Fig. 9 illustrates, two publications explicitly discussed topics related to the make-or-buy decision in AM. Ruffo et al. (2007) distinguished three scenarios: The organisation has no experience in AM, has experience in rapid prototyping, or has experience in rapid or additive manufacturing. For companies with no AM experience at all, implementing AM is high risk, because they are dependent on the knowledge of their suppliers. In this case, making is riskier than buying in additive-manufactured products. Companies that have existing knowledge of rapid prototyping are less dependent on supplier knowledge, and their decisions rely more strongly on production capacity. In-house production with an existing prototyping machine is the least risky option, but this has limited production capacity. The risk in the ‘buy’ option for AM outputs is higher because the dependence on the supplier’s capacity increases. The riskiest option is in-house AM production, where dependence on the knowledge of the suppliers and the required investment are both high. In the case of companies that have already implemented AM, the dependency on suppliers becomes solely capacitive. Here the decision is mostly based on cost. Hedenstierna et al. (2019) performed a quantitative investigation based on a case study. They considered a manufacturing company’s options (make, buy, make and buy) based on the level of specialisation of the relevant AM process and the demand level. In the case of a specialised process at a high level of demand, manufacturing should either take place in-house, or it should be outsourced, whearas a less specialized process at a low-level demand should be outsourced. For a less specialized process at a high level of demand, bidirectional partial outsourcing can be done. This means that the manufacturing of goods is done in cooperation with a partner, resulting in high levels of utilisation of the printer. Where there is a low level of demand for a specialised process, profitable manufacturing becomes infeasible (Hedenstierna et al. 2019).

After a company has decided to outsource certain steps of production, it needs to identify which category of AM it will be requiring. The AM category most frequently addressed (in seven publications) deals with sourcing particular process steps, such as the execution of the manufacturing service by the AM service providers. Rogers et al. (2016) divided AM service providers into three categories: generative services, facilitative services, and selective services. Generative services come before the printing process, such as scanning and construction. When a facilitative service provider is used, the customer already possesses the CAD data and therefore only the printing process itself is outsourced. Selective service providers offer their customers a database from which to choose, and the customer can adapt existing CAD models for printing.

Ruffo et al. (2007) compared the costs of two AM products either manufactured by a service bureau and one manufactured in-house. The make and buy costs of both products decreased slightly by quantity. The result indicated that the self-manufacturing of parts leads to cost advantages. Baldinger et al. (2016) repeated this study in 2016 for buy scenarios with a more diversified set of parts. He found the costs of AM service providers in PBF processes to be comparable to those of in-house production.

Pahwa et al. (2018) introduced a decentralised marketplace for prototyping services, in which designers can upload their CAD data and material specifications. Using an auction-based selection mechanism, which takes suppliers’ capacity, rating and bid price into account, the purchasing task is awarded. Hasan et al. (2013) performed an analysis of interviews and found that AM service providers typically request the CAD data file, the number of parts required, the AM process preferred, and the required material and post-processing steps (e.g. surface finish) before providing a quotation.

Two journal publications focused on the AM input category of CAD data. Hasan et al. (2013) conducted interviews and found that 30% of companies only partly owned the CAD data for the products they sell. Rogers et al. (2016) showed that a design file can either be retrieved from scanning an existing part, making a new construction or downloading the file from a database such as Thingiverse (Friesike et al. 2018). In this way, either the customer, the manufacturing firm or a generative service provider can create the CAD file by themselves or in collaboration. There is no explicit strategy for the acquisition of data.

The procurement of raw material is addressed by two publications (see Fig. 9). Khajavi et al. (2018b) discussed the effects on PBF of the Fray-Farthing-Chan Cambridge process and the production of Ti6AL4V, the most frequently used titanium alloy. This process reduced manufacturing costs and supply chain participants, which minimises the supply risk (Verboeket and Krikke 2019).

Only one publication covered the procurement of 3D printers. Halassi et al. (2018) analysed the motives of consumers who bought 3D printers. The survey revealed that performance expectancy and the price of the printer had no significant influence on the purchasing decision. The purchase was mostly driven by hedonic motivation and the consumer’s ‘do it yourself’ mentality.

## Discussion, future research, implications, concluding remarks and limitations

This paper aimed to present a complete picture of the literature dealing with sourcing in the context of AM. The analysis showed that literature on AM in PSM is scarce. Most articles considered AM in terms of its effects on logistics and SCM, and procurement was rarely addressed. Literature on sourcing in AM is nonexistent. This complete lack of research makes it impossible for this study to identify research gaps in the area of sourcing.

However, drawing on sources in logistics and SCM, the researchers involved in this study are convinced that AM has an effect on industrial manufacturing supply chains. Premises on the ways in which AM affects manufacturing supply chains/logistics will therefore be used to formulate research propositions that outline the effects of AM on sourcing (Table 4). These premises can be used as input when an AM sourcing strategy is being formulated.

**Table 4:** Premises of AM in industrial manufacturing

	AM potentials for manufacturing	Effect on supply (chain)/logistics	Sourcing research fields/research propositions
P1	AM will allow manufacturing to be decentralised	Input goods are needed in a decentralised manufacturing network with fluctuating demands	Establishment of decentralised supply base increases multiple and local sourcing
P2	AM enables the manufacturing of complex goods at low cost	Not only ‘differentiators’ but also companies that follow a cost leadership strategy might produce complex individual goods	Further and increased awareness on initial prices and total costs to strengthen competitive position
P3	AM will decrease manufacturing lead time	Time competition is already established in many industries. This trend will be intensified	Procurement must become agile to establish a fast and flexible supply
P4	AM requires standardised inputs goods for manufacturing	Hitherto structured supply chains (1st tier, 2nd tier) follow the structure of modules, subsystems and systems. This is not necessary per se with AM	Changed market power and negotiation situation compared to the procurement of finished goods
P5	AM transforms the manufacturing of a good into a standardised task, where the processing of the print job becomes unspecific	The question whether to manufacture products in-house or externally becomes blurred and recurring, whereas an agile response is required	Procurement is a manufacturing enabler through manufacturing service contracting
P6	AM requires data as input. Generation or access to data is a core task and precondition for production	Innovation and engineering advantages manifest in data	Procurement must have a more prominent focus on sourcing and securing of data and property rights
P7	AM reduces process steps and components	Physical supply-chain risks are minimised	Procurement integrates AM into concepts that minimise supply side risk
P8	AM has shorter lead times but higher unit costs compared to traditional manufacturing methods	Hedging traditional manufacturing methods with AM is an option to address supply risks	Sourcing can consider AM as an instrument of supplier risk management
P9	AM leads to new or changed business models following industrial platform applications	Supply networks are embedded or at least linked to a number of potential AM platforms	Procurement must develop a sourcing concept for AM platforms and new AM business models

AM allows for the decentralisation of manufacturing (Premise 1, P1) (Mohr and Khan 2015), therefore input goods are required at various locations. This means that demand situations are subject to change (Zhang et al. 2018), which also affects input goods. Consequently, physical AM input goods are required at decentralised manufacturing locations. Because risk must be minimised and changing demand situations demand quick responses, the sourcing concepts of local and multiple sourcing will most likely be applied.

Given that AM allows highly complex products to be manufactured without additional costs (P2) (Durach et al. 2017), even companies that use the competitive strategy of cost leadership (Porter 1985) can produce individualised products without incurring additional costs. This makes the customer more price sensitive, which means that procurement must raise their cost awareness when sourcing input goods.

AM will improve manufacturing lead time (P3). As no tooling is required, products can be manufactured quicker, and the time to market will be shortened (Attaran 2017). This intensifies competition in time to market, as is already the case in various industries. Procurement must therefore enable fast and flexible processes with a supply base that enables a quick reaction to sudden demands.

Input goods in AM become highly standardised (P4). Considering the AM sourcing categories, physical input goods in AM are highly unspecific. Raw material (powder, filament plastics) becomes a standardised commodity (Öberg 2019). A 3D printer is able to execute several designs on different levels of complexity at the same time in the same print job (Durach et al. 2017). The input goods have a large variety of materials (Rogers et al. 2016), but they are highly standardised. Standardisation causes a complexity reduction in supply chains and the elimination of the intermediate tier structures (Mohr and Khan 2015) because functional modules can be printed (Ghadge et al. 2018). These changes increase the negotiation power of procurement as supplier markets develop towards an unspecific “commodity”. Ceteris paribus of the same quality of standardised input goods—the price is a key criterion in the procurement process (see also premise P2).

The processing of the print job in AM becomes a standardised task (P5). The manufacturing process in AM requires little to no supervision (de La Torre et al. 2016). This can confuse the issue of whether to insource or outsource a task, with such concepts as organisationally distributed manufacturing becoming part of the picture (Hedenstierna et al. 2019). The make-or-buy question repeats itself with every new task, which means that more decisions about outsourcing vs insourcing need to be made. In addition, these decisions need to be highly flexible, because they might depend on the availability of free printer capacity (Hedenstierna et al. 2019) and require a fast response. Under such circumstances, procurement needs to enable manufacturing by checking capacities in close relationship with their supplier network, for example AM service providers (Rogers et al. 2016).

Data becomes the key input factor for AM (P6) (Tuck et al. 2007). CAD data can be highly customised (Oettmeier and Hofmann 2016) and topology optimised by bio-inspired algorithms that contain high levels of engineering knowledge (Beyer 2014). CAD data can easily be interchanged because a standardised format (STL) is used (Potter and Eyers 2015). Consequently, procurement must focus on gaining access to data in order to fulfil internal demands, provide data to suppliers and secure sensitive data.

Because AM enables the decentralised (Braziotis et al. 2019) and on-demand (Huang, 2013) printing of functional assemblies (P7) (Ghadge et al. 2018), process steps such as assembly, storage and transportation are reduced. This leads to a more simplified supply chain layout, compared to the global supply chains that we know. The long and complex supply chains of today are exposed to a high number of risks (Wagner and Bode 2007). The reduction in complexity brought about by AM (Mohr and Khan, 2016) would lead to the minimisation (or at least a reduction) of supply chain risk. Procurement might therefore use AM within its sourcing strategy to minimise supply risk.

On the one hand, AM reduces lead times (Muir and Haddud 2018) and makes tooling obsolete (Holmström and Gutowski 2017). On the other hand, AM lacks the advantages of economies of scale (Berman 2012). As a result, for many applications AM is still not price competitive compared to traditional manufacturing methods (P8) (Khajavi et al. 2018a). If it is possible to produce an item with either AM or in the traditional way, AM is usually faster but more costly, while traditional manufacturing is slower but cheaper. From a risk management perspective, AM and traditional manufacturing are opposite risk positions. The ‘hedging’ of traditional manufacturing methods with AM could reduce supply risks by keeping the overall performance high, for example when there is an emergency supply disruption (as during the coronavirus pandemic of 2020). Procurement can therefore consider AM as a risk transition instrument. Developing AM capability makes sense as long as the risk costs in case of supply disruptions are higher than the costs of establishing adequate print capacity to safeguard performance in times of crisis.

Finally, AM leads to new business models and industrial platforms, such as data exchange platforms, engineering service platforms, or even manufacturing platforms (P9). Industrial platforms, which are generally used by companies for coordination and production in a standardised interface (Piezunka 2011), could be used to interconnect buyers and suppliers for data and print-job processing (Potter and Eyers 2015). For procurement, this results in a new steering task and the responsibility to develop appropriate sourcing concepts for these new platforms.

This paper presents a literature review on SfAM and systematises the current basis of scientific knowledge in terms of our analytical framework. Given the hype around AM, it is surprising that there is an almost complete lack of literature on this topic. One explanation for this absence could be that procurement is simply not significantly affected by AM. However, in the discussion section, where we delineate the research proposition by formulating premises on the effects of AM on manufacturing and on supply chain/logistics, we show that AM does in fact have an impact on procurement. P1-6 indicate the changed content requirements of sourcing, while P7-9 indicate completely new sourcing requirements. The latter aspect will be of particular interest for future research on sourcing for AM, especially for the formulation of a holistic sourcing strategy for AM. These premises provide direction for managers in the formulation of a sourcing strategy on AM. Nevertheless, as discussed in the introduction and discussion section, scientific research on PSM and AM lags behind what we know is practically possible. That it is the other way around, managerial practice of AM will provide indications on how sourcing could use AM. During the SARS CoV-2 pandemic in 2020, people around the world have been working on open-access CAD data sets for AM. CAD data is used to manufacture protective face masks and even basic lung ventilators. Print shops across the globe are using CAD data to respond to the urgent demands (Temple 2020). Until now, the observation from the role of AM in the pandemic crisis is in line with the derived premises: Standardised input goods produce highly specific outputs in a decentralised manufacturing network. New business platforms provide data access. AM is more agile and could respond faster. Overall, AM plays a risk mitigation role. It would be of value for further research to explore if and how SfAM can establish a strategy that integrates or hedges AM with traditional manufacturing procedures.

Finally, the authors acknowledge that this research study has several limitations. First, the scope of the literature review is limited to journal articles. Nevertheless, these articles were produced by top academic institutions in the field of procurement. Second, the discussion section is a work in progress and requires further conceptual and theoretical grounding and development. Third, the diffusion of AM is at an early stage, and any assessments, including those proposed in this article, should be treated with caution even when examples of AM in industry and, most importantly, of AM during the pandemic of 2020 indicate that AM will add new agility to strategic thinking in sourcing. Future research should first explore and identify best practice in sourcing for AM. Second, the changes in a company’s susceptibility to sourcing risks in AM should be further analysed to provide an optimal risk position in a portfolio that uses AM and traditional manufacturing collectively.
